# *Intertwined* arbovirus transmission activity: reassessing the transmission cycle paradigm

**DOI:** 10.3389/fphys.2012.00493

**Published:** 2013-01-11

**Authors:** Luis A. Diaz, Fernando S. Flores, Agustín Quaglia, Marta S. Contigiani

**Affiliations:** ^1^Laboratorio de Arbovirus, Instituto de Virología “Dr. J. M. Vanella”, Facultad de Ciencias Médicas, Universidad Nacional de CórdobaCórdoba, Argentina; ^2^Instituto de Investigaciones Biológicas y Tecnológicas, Consejo Nacional de Investigaciones Científicas y Técnicas (IIByT—CONICET)Córdoba, Argentina; ^3^Consejo Nacional de Investigaciones Científicas y Técnicas (CONICET), Ministerio de Ciencia y TecnologíaCórdoba, Argentina

**Keywords:** arbovirus, St. Louis encephalitis virus, transmission cycles, West Nile virus, transmission network

## Abstract

Arboviruses are emerging/reemerging infectious agents worldwide. The factors within this scenario include vector and host population fluctuations, climatic changes, anthropogenic activities that disturb ecosystems, an increase in international flights, human mobility, and genetic mutations that allow spill-over phenomenon. Arboviruses are maintained by biologic transmission among vectors and hosts. Sometimes this biological transmission is specific and includes one vector and host species such as Chikungunya (CHIKV), Dengue (DENV), and urban Yellow Fever (YFV). However, most of the arboviruses are generalist and they use many vectors and hosts species. From this perspective, arboviruses are maintained through a transmission network rather than a transmission cycle. This allows us to understand the complexity and dynamics of the transmission and maintenance of arboviruses in the ecosystems. The old perspective that arboviruses are maintained in close and stable transmission cycles should be modified by a new more integrative and dynamic idea, representing the real scenario where biological interactions have a much broader representation, indicating the constant adaptability of the biological entities.

Arboviruses are emerging/reemerging infectious agents worldwide; Chikungunya (CHIKV), Dengue (DENV), Yellow Fever (YFV), St. Louis encephalitis (SLEV), and West Nile (WNV) are some examples of this phenomenon. Although not fully understood, several factors are thought to promote reemergence. For instance, environmental disturbs from anthropogenic activities (Vasconcelos et al., 2001), climatic changes affecting vector and host population fluctuations (Weaver and Reisen, 2010), human movements through airplanes, animal trade and migration (Pfeffer and Dobler, 2010), and genetic mutations that cause spill-overs (Weaver and Barrett, 2004; Kuno and Chang, 2005).

Basically, arboviruses (arthropod-borne viruses) are maintained by biological transmission through an arthropod vector to a vertebrate host, hence representing an ecological rather than a taxonomic grouping. For most arboviruses (SLEV, Usutu virus—USUV, WNV, Japanese encephalitis virus—JEV, Eastern, Venezuelan, and Western equines encephalitis virus—EEEV, VEEV, WEEV) human beings are dead-end hosts, which means that viremias are not high enough to infect the arthropod vector. Therefore, humans are not necessary for virus maintenance and they represent just an accident during the biological transmission among vectors and hosts. However, CHIKV, DENV, and YFV are exceptions, given that these viruses can replicate and generate viremia titers in the human host high enough to infect vector mosquitoes (Morris, 1988; Scott, 1988; Reisen and Monath, 1989).

Based on ecological terms, infectious agents can be classified as generalist or specialist according to the number of host/vector they can infect. Specialist arboviruses are those transmitted by specific species of host/vector. Thus, as a result of centuries of coadaptation CHIKV, DENV, and YFV are particularly efficient in being transmitted by *Aedes aegypti/Ae. albopictus* mosquitoes and amplified by humans in urban environments (Weaver and Reisen, 2010). In certain cases, some viruses make a change of species and have the ability of being transmitted by another species of host/vector. For example, due to a special mutation, CHIKV is transmitted by an alternative mosquito species: *Aedes albopictus* (Tsetsarkin et al., 2007).

On the other hand, those viruses maintained in nature by more than one host/vector species are considered as generalists, such as: SLEV, WNV, JEV, EEV, WEEV, and VEEV. The analysis of the dynamics maintenance of these viruses is more related to a transmission network than to a transmission cycle.

Several intrinsic and extrinsic requirements (physiological/behavioral—ecological/environmental) must be fulfilled for species to be considered a vector or a host (Table 1). Ecosystems are inherently variable across time and space. The intrinsic characteristics are not modified by time, however, there might be exceptions such as certain selective processes that affect the population, determining susceptibilities to the differential infection among hosts and vectors, e.g., environmental stress and detrimental nutrition affect both vector and host competence (Kramer and Ebel, 2003; Reisen et al., 2003). In contrast, the extrinsic factors such as species availability, density, and abundance are modified in time and space. If we analyze certain ecosystem where the arbovirus is maintained by the network of interactions between its hosts and vectors, we are likely to see that the cycles, part of that network, change through time (Figure 1).

**Table 1:** **Intrinsic and extrinsic characteristics fulfilled by a vector/host of an arbovirus**.

**Characteristics**	**Vectors**	**Hosts**
Intrinsic	Viral replication	
	Susceptibility to viral infection	
	Host feeding preference	–
	Behavior	
Extrinsic	Abundance and dispersal	
	Seasonal breeding patterns	
	–	Attractiveness to mosquito vector
	Host feeding selection	–
	Distribution	

**Figure 1:**
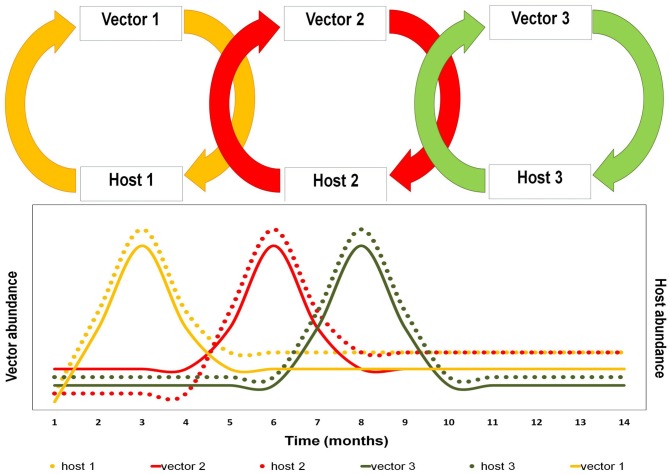
**Schematic representation of the sequencing changes as function in time in arbovirus transmission cycles.** Foot figure: this figure represents the network for a hypothetical arbovirus in which transmission network made by three vector-host cycles. We would like to mention how population fluctuations in vectors and hosts (temporal dynamics) force the virus to adapt itself in relation to availability of vectors and hosts.

In systems like the arbovirus, characterized by multi-host-vector interactions, the ecological dynamics may alter the epidemiological patterns and scenarios (Allan et al., 2009). Many studies have recently focused on the effects of biodiversity over arbovirus activity (Ezenwa et al., 2006, 2007; Swaddle and Calos, 2008; Allan et al., 2009; Loss et al., 2009; McKenzie and Goulet, 2010). It has been hypothesized that high diversity of host would result in a reduced viral activity. This could be caused by two different mechanisms, firstly, due to a decrease in the probability that the vector comes into contact with the host of higher competence; resulting this out of a decrease in the relative abundance of the host with the higher competence or an increase in the relative abundance of hosts of low competence. Secondly, higher host diversity could increase interspecific interactions, such as predation and competition, thus potentially regulating the abundance of the most competent host (Allan et al., 2009).

Empirical studies aimed to test the hypothesis that higher host diversity reduces transmission rate and viral concentration in the ecosystem had produce inconclusive results. While some studies have found support for this hypothesis (Ezenwa et al., 2006; Allan et al., 2009), others have not found a relation between host diversity and virus transmission (local spatial scale vs. regional/national) (Loss et al., 2009). Moreover, diversity effects on transmission dynamics vary through time. For instance, by using a country scale analysis, Allan et al. (2009) found that the fluctuation of WNV incidence in the American population between 2002 and 2004 is explained through bird diversity, while other factors such as human density and the community competence index vary in their relevance during the years. These studies emphasize the need to consider the diversity of host and vector within an ecosystem when analyzing virus dynamics (Kilpatrick, 2011). Some authors pointed out the hypothesis that host competence could be associated to evolutive relatedness (Ezenwa et al., 2006). Kilpatrick et al. (2007) showed that host competition varies more among families than within members of the same family. Therefore, when assessing the suitability of an ecosystem to an arbovirus it is important to know and consider not only the diversity (abundance, richness) but also the species composition of potential vectors and hosts.

Besides its geographical distributions, the virus adaptability for its maintenance also occurs at a seasonal level, being the viral flow driven by the feeding preference of the vectors (Kilpatrick et al., 2006b). Thus, the strength level of a certain host/vector association can be quantified by measuring the vector-feeding preference. Nowadays, thanks to the incorporation of molecular techniques (e.g., gene sequencing for Cytochrome Oxidase I), the vectors blood-feeding patterns can be identify (Apperson et al., 2002; Goldstein and DeSalle, 2011). Based on both blood-feeding patterns and host population densities, a feeding selection index can be determined and later translated into a host-vector association measurement (Manly et al., 2002; Hamer et al., 2011). This feeding preference is modified by extrinsic factors such as host abundance, vector densities, and/or avian defensive behavior (Kilpatrick et al., 2006b; Molaei et al., 2006; Thiemann et al., 2011). For instance, in late summers at a countrywide scale in the USA, WNV vectors (*Cx. pipiens, Cx. nigripalpus, Cx. tarsalis, Cx. salinarius*) showed a shift in their feeding preference from birds to mammals (Kilpatrick et al., 2006b). Although the impact of genetic it not yet explored, physiological or other intrinsic changes within the mosquito population may contribute to this host shift.

As empirical examples we here reconsider the transmission pattern of WNV and SLEV in the American continent, mainly based on the data gathered in USA and Argentina (Figures 2A,B).

**Figure 2:**
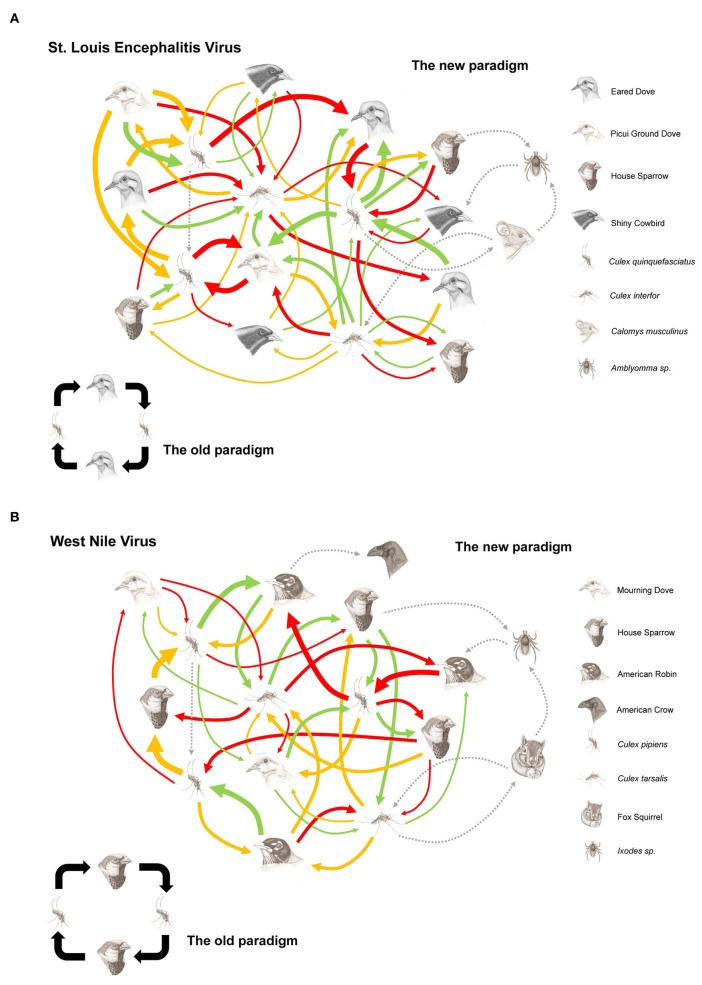
**Hypothetical transmission networks for St. Louis encephalitis virus in central area Argentina (A) and West Nile virus in USA (B).** Foot figure: The arrows represent the viral flow between the vectors and hosts involved in the arbovirus maintenance network. The thickness of the arrow represents the amount of existing virus between the particular connection of host and vector (which is determined by the vector host preference, vector-host population density, vector and host competence). The spotted line arrows represent alternative transmission way (venereal and/or transovarial transmission), hosts (mammals) and vectors (ticks). The colored arrows represent the season in which the vector-host relation takes place (green: Spring, red: Summer, orange: Fall).

Since its introduction in 1999 in the USA, WNV has become one of the arboviruses of most medical concern in the American continent. Thanks to a decade of ecological and epidemiological research carried out in the USA, most aspects of its transmission dynamics have been analyzed. WNV is maintained through biological transmissions in which *Culex* spp. mosquitoes are involved as vectors and Passeriformes birds as hosts (Komar et al., 2003; Hayes et al., 2005). The main species of vector are *Cx. pipiens, Cx. restuans* (Kilpatrick et al., 2005), *Cx. quinquefasciatus* (Turell et al., 2005), *Cx. nigripalpus* and *Cx. tarsalis* (Turell et al., 2002; Blackmore et al., 2003); while only a few non-*Culex* species have been considered as possible vectors, such as *Aedes albopictus* and *Ae. vexans* (Turell et al., 2005). Regarding hosts, the main participants could be listed as: the American Robin (*Turdus migratorius*), the Northern Cardinal (*Cardinalis cardinalis*), the House Sparrow (*Passer domesticus*), the Blue Jay (*Cyanocita cristata*), the Northern Mockingbird (*Mimus polyglottos*), the Western Scrub-Jay (*Aphelocoma californica*), the American Crow (*Corvus brachyrhynchos*), and the Black-billed Magpie (*Pica hudsonia*) (Kilpatrick et al., 2007). Geographical and seasonal variation in host and vectors were observed across the USA. In the northeastern region of USA, the suggested main vectors are *Cx. pipiens* and *Cx. restuans* (Kilpatrick et al., 2005), while *Cx. salinarius* might have local significance as a bridge vector (Molaei et al., 2006). The American Robin has been suggested to be the main host in this region (Apperson et al., 2002, 2004), as well as in Tennessee (Savage et al., 2007) and in the mid-eastern region of the country (Kilpatrick et al., 2006a; Griffing et al., 2007). Additionally, the Northern Cardinal, the House Finch, and the House Sparrow are suggested to be important hosts (Molaei et al., 2006) for the region. The most important vector in Florida are *Cx. nigripalpus* and *Cx. quinquefasciatus* (Sardelis et al., 2001; Goddard et al., 2002; Rutledge et al., 2003); and in terms of hosts, the Northern Cardinal, the House Sparrow, the Blue Jay, and the Northern Mockingbird were mentioned for this state (Komar et al., 2005). In the western area of USA the principal vector species are *Cx. tarsalis, Cx. quinquefasciatus* and *Cx. stigmatosoma* (Goddard et al., 2002; Reisen et al., 2005). Birds species suggested as host are the House Finch, the House Sparrow, the Western Scrub Jay (*Aphelocoma californica*), the Mourning Dove (*Zenaida macroura*), and the Common Ground Dove (*Columbina passerina*) (Reisen et al., 2005).

*Cx. erraticus, Coq. perturbans*, and *Cx. salinarius* may play a more significant role in the transmission of WNV in the USA mid-southern region (Cupp et al., 2007)*.* Other potential host competences are the Western Scrub-Jay, the American Crow, the Black-billed Magpie, the Common Grackle (*Quiscalus quiscula*), the House Finch, and the Ring-billed Gull (*Larus delawarensis*) (Kilpatrick et al., 2007). WNV mosquito vectors show seasonal variation across their geographical distribution. *Cx. restuans* is generally found in spring and early summer, while other *Culex* species are present later in the season (O'Meara et al., 1989; Andreadis et al., 2001; Ebel et al., 2005). The *Cx. pipiens/restuans* complex (not differentiated due to morphological similarity) responded differently to weather variables in western New York (USA) than another potential WNV vector, *Ae. vexans* (Trawinski and Mackay, 2008). Conversely, *Cx. pipiens* and *Cx. quinquefasciatus* showed similar seasonal distributions in Tennessee (USA), though *Cx. quinquefasciatus* had a broader seasonal distribution and there was variation between sites (Savage et al., 2008). In some locations, since *Cx. salinarius* frequently feeds on both birds and mammals (Kilpatrick et al., 2005) it could also be an important epidemic or bridge vector (Andreadis et al., 2004).

Alternative transmissions mechanisms contribute to the maintenance of WNV in nature. For instance, in vertical vector transmission the virus is transmitted to its descendant through an infected female mosquito. This way tends to have low transmission rates, but gains importance in mild areas as an overwinter mechanism. Laboratory and field studies have confirmed that WNV can be vertically transmitted in at least *Culex* and *Aedes* mosquitoes (Anderson and Main, 2006; Unlu et al., 2010). Other alternative mechanism is the direct transmission among hosts. This mechanism has been observed in several species of birds maintained under laboratory conditions, where viruses transmissions occurs both, due to the intake of WNV-infected food [e.g., Great Horned Owl (*Bubo virginianus*), American Crow, Common Grackle, House Finch and House Sparrow, or direct transmission between partners (Ring-billed Gull, Blue Jay, Black-billed Magpie, and American Crow) (Komar et al., 2003)]. Apart from mosquitoes and birds being the key participants in the maintenance of the virus in nature, other animals such as fox squirrels (*Sciurus niger*) (Root et al., 2006) and ticks (*Dermacentor andersoni, D. variabilis, Ixodes scapularis*) (Anderson et al., 2003) might play a fundamental role.

A second example where the maintenance of an arbovirus in nature exceeds the vector-host cycle model of transmission is the SLEV flavivirus. This multi-host-vector virus is a reemerging close relative of WNV and JEV, and it is exclusively distributed in the American continent. SLEV is maintained through the biological transmission among *Culex* spp. mosquitoes and Passeriformes and Columbiformes hosts (Reisen, 2003). Since many avian host and *Culex* mosquito vector species can transmit this virus, it can be consider as a generalist. For example, in California, at least three species of *Culex* mosquitoes (*Cx. tarsalis, Cx. stigmatosoma*, and *Cx. quinquefasciatus*) can transmit the virus to different passerines birds (House Sparrow, House Finch). However, in most part of the USA (center and eastern) it is mainly maintained by the House Sparrow and *Culex quinquefasciatus*. In contrast, in Argentina the House Sparrow has an insignificant role in the maintenance of the virus in nature. Host competence studies have shown that in Argentina the Eared Dove (*Zenaida auriculata*) and the Picui Ground Dove (*Columbina picuí*) are the principal amplifying hosts. However, despite their lower viremia, other species (the House Sparrow, the Spotted Winged Pigeon—*Patagioenas maculosa*, the Shiny Cowbird—*Molothrus bonariensis*, the Bay Winged Cowbird—*Agelaioides badius*), replicate the virus with a titer high enough to infect the *Cx. quinquefasciatus* vector (Diaz, 2009). With regards to vectors, SLEV has been found in the mosquitoes *Ae. aegypti, Ae. albifasciatus, Ae. scapularis, An. albitarsis, Cx. apicinus, Cx. interfor, Cx. quinquefasciatus, Psorophora ferox* (Díaz et al., 2012). However, certain field and laboratory assays strongly suggest that *Cx. quinquefasciatus* and *Cx. interfor* are the main SLEV vectors (Diaz et al., 2006; Diaz, 2009).

As seen for WNV, vertical transmission has been observed for SLEV. This mechanism could explain the permanence of the virus in nature (Flores et al., 2010; Díaz et al., 2012). A hypothetical alternative transmission cycle for SLEV can be thought to exist between *Calomys musculinus* and *Mus musculus* rodents and mosquitoes such as *Ae. albifasciatus, Ae. scapularis*, and *Cx. quinquefasciatus* (Sabattini et al., 1998; Diaz, 2009).

Resuming, the scheme of biological interaction between vectors, host, and viruses is complex. These intricate networks of interactions would favor a given virus with a greater stability against biological and adverse environmental conditions (e.g., population declaims of one of host and vector). The existent strength of viral-flow cycles between the vectors and host conforming the network (or degree of association) are affected by environmental and climatic factors that vary in time and space (Figures 1 and 2). Given the inherent complexity of the vectorial transmission among hosts, it is necessary to carry out ecological and epidemiological research for each epidemical event.

The mechanisms for the maintenance of generalist arboviruses are inherently complex, and should be taken into account, and incorporated, when designing and constructing mathematical models that allow us to predict its enzootic/epidemic activity in a given ecosystem (wild, urban, agricultural, etc.). By facing these new designs from an integral, networkable perspective we can improve our predictive ability. The ultimate goal will be to improve our understanding of viruses' dynamics and thus improve preventive measures in vector-control and public health policies.

## In conclusion

Arbovirus maintenance in nature depends on host and vector coexistence in time and space. From an ecosystemic approach of transmission cycles, it is unlikely that the maintenance of a virus is restricted to determined vectors and hosts, particularly when considering a multi-host-vector virus, like certain arboviruses (e.g., SLEV, WNV). Therefore, the determining factors for the maintenance of an arbovirus in nature are the intertwined biological links that integrate a transmission network rather than a transmission cycle. Consequently, to better understand the activity pattern and transmission networks of arboviruses, it is fundamental to understand the species assembly of hosts and vectors, their interactions, and fluctuation through time and space.

### Conflict of interest statement

The authors declare that the research was conducted in the absence of any commercial or financial relationships that could be construed as a potential conflict of interest.
